# Biotype Determines Survival of *Yersinia enterocolitica* in Red Blood Cell Concentrates

**DOI:** 10.3390/ijms26125775

**Published:** 2025-06-16

**Authors:** Katarzyna Morka, Sylwia Banaszkiewicz, Jakub Korkus, Jacek Bania, Jarosław Bystroń, Gabriela Bugla-Płoskońska, Marta Stanek, Urszula Sokalska, Małgorzata Szymczyk-Nużka, Samuel K. Sheppard, Ben Pascoe

**Affiliations:** 1 Department of Food Hygiene and Consumer Health Protection, Wrocław University of Environmental and Life Sciences, Norwida Str. 31, 50-375 Wrocław, Poland; sylwia.banaszkiewicz@upwr.edu.pl (S.B.); jakub.korkus@upwr.edu.pl (J.K.); jacek.bania@upwr.edu.pl (J.B.); jaroslaw.bystron@upwr.edu.pl (J.B.); 2 Department of Microbiology, Faculty of Biological Sciences, University of Wroclaw, Przybyszewskiego 63 St., 51-148 Wrocław, Poland; gabriela.bugla-ploskonska@uwr.edu.pl; 3 Regional Center for Blood Donation and Treatment, Czerwonego Krzyża 5/9, 50-345 Wrocław, Poland; marta.stanek@rckik.wroclaw.pl (M.S.); urszula.sokalska@rckik.wroclaw.pl (U.S.); malgorzata.szymczyknuzka@rckik.wroclaw.pl (M.S.-N.); 4 Ineos Oxford Institute for Antimicrobial Research, Department of Biology, University of Oxford, Mansfield Road, Oxford OX1 3RB, UK; samuel.sheppard@biology.ox.ac.uk (S.K.S.); ben.pascoe@ndm.ox.ac.uk (B.P.)

**Keywords:** *Yersinia enterocolitica*, bioserotypes, blood safety, contamination, red blood cells

## Abstract

Red blood cell (RBC) concentrates remain at risk of bacterial contamination during cold storage. Although infrequent, *Yersinia enterocolitica* poses a significant blood safety risk. This study aimed to assess *Y. enterocolitica* bioserotype growth in RBC concentrates, serum sensitivity, and genetic diversity including iron metabolism genes. Ten *Y. enterocolitica* isolates from bioserotypes 1A, 1B/O:8, 4/O:3, and 2/O:9 were incubated in RBC concentrates and counted on days 3, 7, 14, 21, and 28. After incubation, the isolates were tested in human serum (NHS). Eight genomes were sequenced, analyzed using cgMLST, and screened for iron metabolism genes. The isolates formed two clusters, with 186dz (1A) and Ye8 (1B/O:8) as singletons. After 28 days in the RBC concentrates, the bacterial counts ranged from 1.98 × 10⁵ to 1.2 × 10⁹ CFU/mL, with Ye8 (1B/O:8) achieving the highest growth and one 4/O:3 isolate showing the lowest. All isolates survived 15–30 min in NHS, but the 28s isolate did not survive at 60 min. Serum sensitivity increased in two isolates, decreased in three, and remained unchanged in five. Isolates contained 27–42 iron metabolism genes with multiple allelic variants. The iron metabolism gene content or variants may influence the growth of *Y. enterocolitica* in RBC.

## 1. Introduction

The transfusion of red blood cell (RBC) concentrates is the most common medical procedure and is often a life-saving intervention for patients [1]. RBC concentrates are not routinely tested for the presence of microorganisms [2]. The transfusion of microbiologically contaminated RBC concentrates is a rare phenomenon, but may lead to severe and even fatal complications for patients. Infections can arise from commensal skin bacteria as well as bacteria like *Yersinia enterocolitica* or other cold-tolerant organisms that may go undetected in donors with transient bacteremia, possibly due to recent intestinal yersiniosis [1,3]. Research by Prax et al. [4] demonstrated that even under refrigerated storage, *Y. enterocolitica* could proliferate extensively in RBC concentrates. Other psychrotrophic pathogens, such as *L. monocytogenes*, *S. liquefaciens*, *S. marcescens*, and *P. fluorescens* can also multiply in stored RBC concentrates. For most of these psychrotrophs, the key is an iatrogenic source of contamination such as a colonized hospital environment, blood bags, or test tubes. However, for *Y. enterocolitica*, the blood donor themselves can serve as a direct source of RBC infection [1,5,6,7].

Up to 46% of sepsis cases following RBC concentrate transfusion are caused by *Y. enterocolitica*, 25% by *Pseudomonas* spp., 11% by *Serratia* spp., and 18% by other bacteria [1,8]. An analysis of 55 cases of post-transfusion sepsis caused by *Y. enterocolitica* between 1975 and 2007 showed an overall mortality rate of 54.5% among RBC concentrate recipients. Among the donors responsible for these infections, 89.5% had antibodies for *Y. enterocolitica*, indicating prior exposure, and 51% reported gastrointestinal symptoms a month before or during blood donation [1]. These cases of post-transfusion yersiniosis typically originate from blood donors who had recently experienced a self-limited diarrhea followed by asymptomatic bacteremia.

The first documented case of transfusion-related yersiniosis was published in the Netherlands in 1975 [9]. More recent cases include a 2015 report of a woman who developed fatal septicemia caused by *Y. enterocolitica* serotype O:9 a few days after childbirth [7]; a 2007 case involving a 23-year-old man who developed hemorrhagic shock and multi-organ failure following an RBC concentrate transfusion for recurrent pneumothorax [5]; and a fatal case in a 71-year-old diabetic patient with anemia after heart surgery, infected by *Y. enterocolitica* bioserotype 4/O:3 contaminating the RBC concentrate [10]. Symptoms that appeared after RBC concentrate transfusion included fever, chills, abdominal pain, cyanosis, and acute respiratory distress syndrome, ultimately leading to death [10]. Autotransfusion of the patient’s own blood can also be a source of infection, as seen in a 13-year-old patient who had previously experienced mild diarrhea and received four units of RBC concentrate that had been collected before surgery. After transfusion, the patient developed symptoms of fever, low blood pressure, and metabolic acidosis with reduced serum bicarbonate levels [11]. This case of septic shock due to autologous RBC concentrate transfusion highlights *Y. enterocolitica*’s potential to cause bacteremia and subsequently proliferate in blood components [11].

To mitigate the risk of post-transfusion yersiniosis, several measures have been proposed [10]. First, blood donors should be thoroughly screened for recent gastrointestinal infections, particularly within 3.5 to 6 months before donation. However, this may result in excluding many donors, potentially limiting the blood supply. Another measure is to shorten the RBC storage duration from 42 days to 21–25 days, as older RBC units are more commonly associated with transfusion-related yersiniosis. Additionally, testing blood components for *Y. enterocolitica* antibodies has been suggested to identify potential infections among donors before donation, although this would increase the costs. A standard practice in many European countries is leukodepletion—removing white blood cells from blood components using special filters, which can reduce the infection risk by around 80%. Bacteriological testing of blood components is another proposed solution, although it is the most effective, it is also costly and time-consuming [10].

In Poland, the current guidelines from the Ministry of Health (30 March 2021) state that RBC concentrate preparation should be completed in a single stage, immediately after donation. This process includes centrifugation, separation, visual inspection, and removal of the buffy coat. It must follow validated procedures to minimize microbiological contamination and bacterial growth in blood components [12]. While routine bacteriological testing of blood components is not performed, validation of the process with sterile welding machines and the disinfection of donor skin before blood collection is mandatory. Donors are temporarily disqualified from donating blood for at least two weeks following recovery from diarrhea, and for 28 days after full recovery from yersiniosis [12].

*Y. enterocolitica* consists of six biotypes (1A, 1B, 2, 3, 4, 5) that differ in their level of pathogenicity against humans. The main source of yersiniosis infection in humans is pigs; however, wild boars (*Sus scrofa*) have recently become an important new reservoir for *Y. enterocolitica*, partly due to the increasing popularity of game meat consumption. To date, differences in the ability of *Y. enterocolitica* to survive in RBC concentrates of different biotypes have not been studied. In the literature, there are reports about *Y. enterocolitica* growth in RBC concentrates, however, the variables examined there concerned RBC concentrates, their preservation buffers, exposure time to room temperature, and type of preparation rather than specific details about the microorganisms [2,4,13].

*Y. enterocolitica* can grow in blood and blood components due to its resistance to human serum and its ability to multiply at refrigerated temperatures (1–6 °C) [13]. The anticoagulant-preservative buffer and additive solutions of RBC concentrates contain glucose and adenine, which are a source of carbon and energy for the growth of *Y. enterocolitica*. Moreover, a pH = 7.3 of RBC concentrate is optimal for the growth of *Y. enterocolitica* [1]. During RBC concentrate storage, iron is released from breaking down blood cells [14]. Changes in iron concentration in the environment affect the growth and cellular metabolism of bacteria. There is an assumption that the existence of specific iron acquisition-related genes may be responsible for some of the differences in the survival of *Y. enterocolitica* strains in RBC concentrates [15,16]. Yersiniabactin (Ybt) is the intrinsic endogenous siderophore iron uptake system in the highly virulent *Y. enterocolitica* 1B biotype, encoded by the *ybt* gene cluster (*irp1-9*, *fyuA*) on High Pathogenicity Island (HPI) [17,18]. Ybt binds Fe^3+^ with high affinity and the yersiniabactin–iron complex is then transported into the bacterial cell. Alternative iron-scavenging systems potentially utilized by biotypes other than 1B include the uptake of the enterochelin (enterobactin) catecholate siderophore, which is widely distributed among *Enterobacteriaceae*. *Y. enteorocolitica* possesses the genes *fepBDGC*, *fepA*, and *fes*, which are involved in this uptake but are unable to synthesize enterochelin itself [19]. Another iron sequestration mechanism involves the heme uptake systems (*Hem* and *Has*), encoded by *hemRSTUV*, *hasA*, and *hasR*, which enable the uptake of heme and hemoglobin as iron sources. The TonB-dependent OM transporter/receptor system encoded by *fecA*, *fepA*, *foxA*, and *fcuA* facilitates the acquisition of foreign siderophore–Fe^3+^ complexes. The Feo system, which comprises the *feoABC* gene cluster, mediates the transport of ferrous iron (Fe^2+^) [15,16,20].

*Y. enterocolitica* is classified into six biotypes (1A, 1B, 2, 3, 4, and 5) and approximately 70 serotypes, based on the structural variations of the lipopolysaccharide O-antigen [21]. The primary difference between *Y. enterocolitica* biotypes is based on distinct biochemical and phenotypic characteristics described by Wauters et al. [22]. This classification corresponds with geographical distribution and is associated with specific clinical manifestations [23]. Furthermore, biotypes can be differentiated at the genetic level, particularly through variations in virulence factors: biotypes 2, 3, 4, and 5 harbor the pYV plasmid, biotype 1B also carries the HPI, whereas biotype 1A lacks both of these virulence determinants. Only the 1B and 2–5 biotypes possess pathogenicity-related genes such as *ail* and *yadA,* which confer resistance to human serum complement and promote systemic dissemination [24]. Until recently, this classification suggested that biotype 1B is highly virulent, biotypes 2–5 are weakly pathogenic, and biotype 1A is non-pathogenic; however, recent findings indicate that some strains within biotype 1A may possess pathogenic potential, partly attributed to the acquisition of the *ail* gene [25,26,27,28]. However, to accurately assess the potential function of the *ail* gene in biotype 1A, it is necessary to analyze its sequence variants—specifically A4 to A6—which are typical of non-pathogenic biotype 1A strains and contain missense mutations [26]. Biotype 1A compensates for the absence of classical virulence factors by expressing alternative virulence-associated genes encoding the pore-forming toxins YaxA and YaxB, *invA*, *ystB*, the adhesion factor *MyfA*, the enterochelin utilization gene cluster (*fepBDGC*, *fepA*, *fes*), and, in some strains, the insecticidal toxin complex gene whose role in *Y. enterocolitica* pathogenesis remains unelucidated [29,30]. These observations challenge the established classification of *Y. enterocolitica* biotypes and the corresponding assumptions about their pathogenicity.

This study examined *Y. enterocolitica* isolates belonging to the strongly pathogenic bioserotype 1B/O:8, weakly pathogenic bioserotypes 4/O:3 and 2/O:9, and isolates of bioserotype 1A considered as non-pathogenic. Isolates tested in this study were genotyped using cgMLST (core-genome multilocus sequence typing) and the repertoire of iron metabolism-related genes was determined based on genomic data. *Y. enterocolitica* isolates were grown in RBC concentrates, then their sensitivity to exposition to human serum was tested. The aim of this study was to assess whether a bioserotype of *Y. enterocolitica* or iron acquisition and storage-related genes may be linked to the dynamics of bacterial growth in RBC concentrates. We also aimed to assess whether prolonged incubation in RBC concentrates affects the sensitivity of *Y. enterocolitica* to human blood serum, assuming that such incubation could lead to bacterial adaptation to the conditions, which would subsequently enable it to better survive in serum after transfusion to the recipient.

## 2. Results

### 2.1. Whole-Genome Sequencing of Y. enterocolitica Isolates

Whole-genome sequencing of eight *Y. enterocolitica* isolates resulted in obtaining genome sequences with a mean length of 4,590,698 bp (Supplementary Table S1). The number of contigs ranged from 197 to 412 and the GC (%) content was 47.13 ± 0.19. Genomic sequences of the studied isolates were deposited at the NCBI database under BioProject accession number PRJNA1144334. All contigs under 200 bp in length were manually removed.

### 2.2. Phylogenetic Analysis and Detection of Iron Metabolism Genes in Y. enterocolitica Isolates

**Figure 1:**
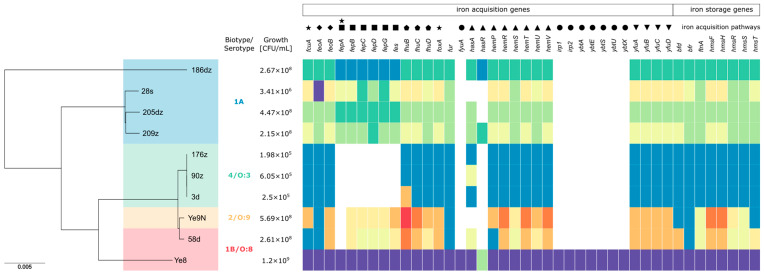
Phylogenetic tree of the studied *Y. enterocolitica* isolates, alongside the distribution of 42 iron metabolism-related genes and their growth potential in red blood cells (RBCs) after 28 days of incubation. The tree was constructed using the core genome multilocus sequence typing (cgMLST) scheme and the Jukes and Cantor evolutionary model, visualized with Phandango. Each isolate is annotated with its corresponding iron metabolism-related gene repertoire. Genes are represented by colored blocks indicating different allelic variants, while white blocks signify gene absence. Clades in the phylogenetic tree highlight genetic relationships among isolates including differences in bioserotypes, allelic repertoires, and the presence or absence of specific genes. The number of detected genes in each isolate ranged from 27 to 42, reflecting variation in their iron acquisition capabilities. Reference strains Ye8 and Ye9N are included for comparison, with Ye8 harboring all 42 genes. Clustering patterns correlate with specific genetic characteristics. Legend:●—yersiniabactin (Ybt), ∎—enterochelin catecholate siderophore, ▲—Hem uptake system, ★—TonB-dependent OM transporters, ◆—Feo system, ▼—inorganic iron ABC transport systems of *Y. pestis* (Yfu), ⬟—ferric hydroxamate uptake system (*fhuCDB*), *fur*—the master regulator of iron homeostasis in *Y. enterocolitica*, *bfr*—bacterioferritin, *bfd*—bacterioferritin-associated ferredoxin complex, *ftn*—ferritin, *hmsFRST*—hemin storage system.

The relationship between eight *Y. enterocolitica* isolates and two *Y. enterocolitica* reference strains was investigated through construction of a neighbor-joining phylogenetic tree constructed from an alignment of cgMLST genes (*n* = 42). Clustering by cgMLST generally matched the bioserotypes. Most of the isolates formed two clusters, while isolate 186dz and the Ye8 strain formed their own singleton branches. Isolates 28s, 205dz, and 209z belonging to the 1A bioserotype clustered together, but a fourth 1A serotype, isolate 186dz, was found to be genetically distant. In turn, all of the 4/O:3 isolates (i.e., 176z, 90z, and 3d) clustered together according to cgMLST. The 58d isolate and Ye8 reference strain, both belonging to the 1B/O:8 serotype, were more distantly related than the 58d isolate and Ye9N strains, the last classified as the 2/O:9 serotype (Figure 1).

Genomes of all sequenced isolates, along with the Ye8 and Ye9N *Y. enterocolitica* reference strains were screened for the presence of 42 iron metabolism-related genes. The presence of 27 to 42 genes was found in all of the tested isolates. Each of the studied genes responsible for iron metabolism occurred in numerous allelic variants (Figure 1). The content of iron metabolism related-genes was similar in isolates 186dz, 28s, 205dz, and 209dz, but the *hasA* gene was absent from isolate 28s. Each of the above isolates carried a specific allele repertoire of iron metabolism related-genes. Isolates 90z, 176z, and 3d harbored 27 iron metabolism related-genes of the same allelic variants, except the *hasA* gene, having a different variant in the 90z isolate. This group of isolates, representing the 4/O:3 biotype and being genotypically closely related, lacked 15 iron acquisition genes when compared to the remaining isolates. The Ye8 isolate harbored all 42 of the studied iron metabolism related-genes. The genomes of isolates 58d and Ye9N, classified as different bioserotypes but closely related genetically, contained 33 and 32 of the studied genes, respectively, with 12 genes sharing the same allelic variant.

### 2.3. Growth of Y. enterocolitica in the RBC Units

**Figure 2:**
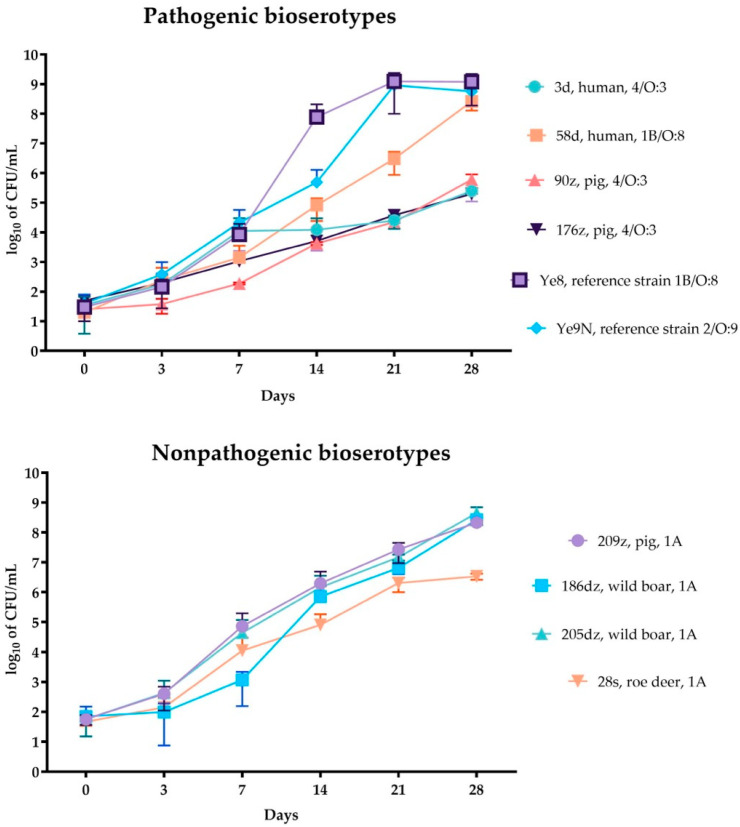
Bacterial growth in triplicates in RBC concentrates during 28 days of incubation at 2–4 °C. The data from each RBC incubation were compared by analysis of variance (two-way ANOVA). Bacterial survival rates were compared using the Tukey’s multiple comparisons test. The *p*-values from these tests are shown in the text. *p* < 0.05 indicates that the compared values were significantly different at a 95 % confidence level.

All of the investigated *Y. enterocolitica* isolates were able to grow in RBC concentrates (Table 1). The increase in bacterial count was observed starting from the third day after inoculation. The highest bacterial count after 28 days of storage in the RBC concentrates was observed for the reference 1B/O:8 *Y. enterocolitica* strain Ye8 (1.20 × 10^9^), and the lowest CFU/mL value was achieved by the 4/O:3 *Y. enterocolitica* isolate 176z (1.98 × 10^5^) (*p* < 0.05, Table 1, Figure 2). The 1A biotype isolates 209z, 186dz, and 205dz reached similar CFU/mL values after 28 days of incubation (2.15 × 10^8^, 2.67 × 10^8^, 4.47 × 10^8^ CFU/mL, respectively), while the other 1A biotype 28s isolate, genetically clustering with the isolates 205dz and 209z, reached 3.41 × 10^6^ CFU/mL at 28 days (*p* < 0.05, Figure 2).

The *Y. enterocolitica* Ye9N strain and 58d isolate produced a similar bacterial count after 28-days of RBC concentrate incubation (5.69 × 10^9^ and 2.61 × 10^8^ CFU/mL, respectively) (Figure 2).

Isolates belonging to the 4/O:3 bioserotype (i.e., 176z, 90z, and 3d), genetically clustered together, had a comparable count at 28 days of RBC concentrate incubation, being in the range of 1.98 × 10^5^ to 6.05 × 10^5^ CFU/mL) (*p* < 0.05, Table 1, Figure 2).

The most statistically significant differences between isolates were observed only after 21 days of storage in RBC concentrate, when the *Y. enterocolitica* reference 1B/O:8 strain demonstrated significantly greater growth compared with isolates 3d, 58d, 90z, 176z, 186dz, and 28s (*p* = 0.0004) as well as 209z (*p* = 0.0006). After 28 days of storage, statistically significant growth differences were noted only between the *Y. enterocolitica* reference 1B/O:8 strain and isolates 3d, 90z, 176z (*p* = 0.0008), 209z (*p* = 0.0314), and 28s (*p* = 0.0009).

### 2.4. Pathogenic Bioserotypes Survived Longer Exposure to NHS

**Figure 3:**
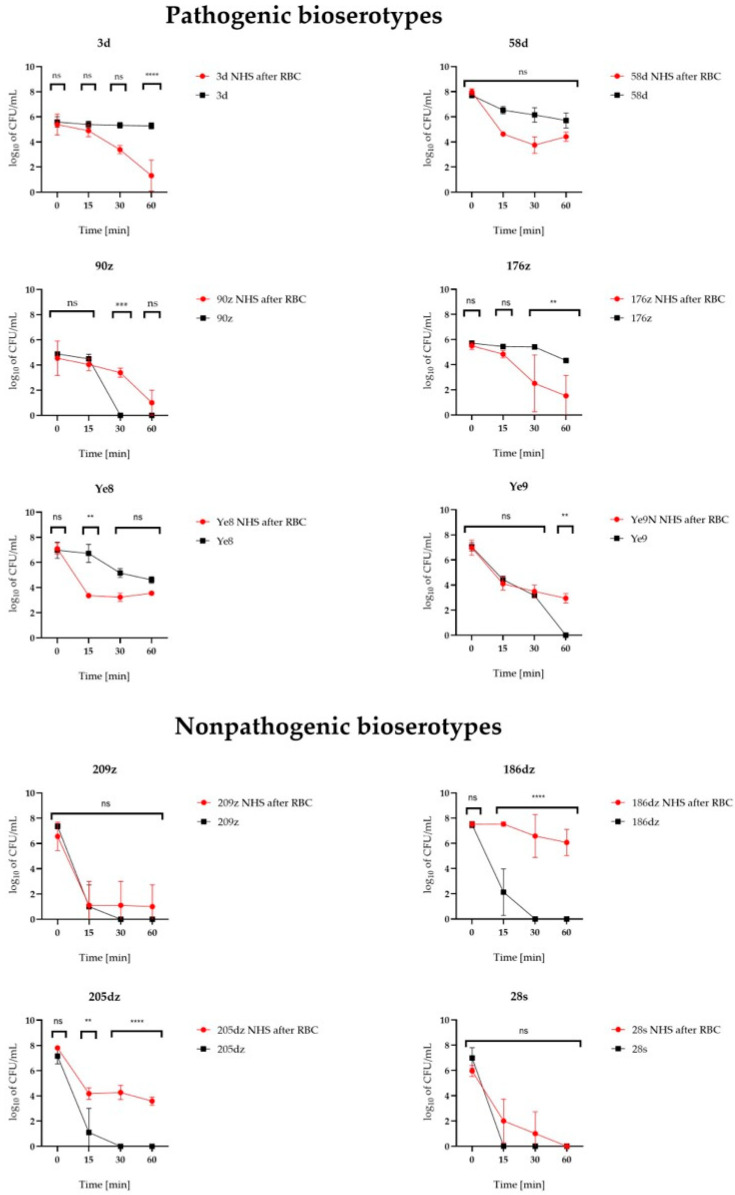
Survival of pathogenic and nonpathogenic 1A *Y. enterocolitica* isolates during 60 min at 37 °C in NHS before and after RBC concentrate incubation. The asterisks indicate statistically significant differences between strains (ns *p* > 0.05, ** *p* < 0.01, *** *p* < 0.001, **** *p* < 0.0001, by two-way ANOVA with Tukey’s comparison post-test).

The effect of exposure to NHS on the survival of *Y. enterocolitica* was assessed. All of the isolates kept in control conditions (not exposed to RBC), except for the 28s *Y. enterocolitica* isolate, survived the 15 min exposition on NHS (*p* < 0.0001). Exposure of *Y. enterocolitica* to NHS for longer periods of 30 and 60 min led to a significant decrease in the bacterial counts (*p* < 0.0001). Only five and four out of the ten isolates kept in control conditions survived the 30 min (3d, 58d, 176z, Ye8, Ye9) and 60 min (3d, 58d, 176z, Ye8) NHS exposition, respectively. All of these isolates belonged to bioserotypes considered as pathogenic. None of the isolates belonging to the non-pathogenic 1A biotype survived the 30 min exposition to NHS (*p* < 0.0001, Figure 3, Table 2).

All strains after 28-days of RBC concentrate incubation survived the 15 min, 30 min, and 60 min exposition on NHS, however, the 28s *Y. enterocolitica* isolate did not survive 60 min (*p* < 0.0001, Figure 3, Table 2).

Comparison of NHS survival between the control and RBC-grown *Y. enterocolitica* isolates at 60 min indicated that the 3d and 176z isolates became more sensitive to human serum after RBC concentrate incubation (*p* < 0.0001, and *p* = 0.006, respectively). The *Y. enterocolitica* 3d isolate exhibited a bacterial count of 2.14 × 10^5^ CFU/mL after 60 min of incubation in NHS without prior exposure to the RBC concentrate. However, following incubation in RBC concentrate, this value decreased markedly to 1.1 × 10^2^ CFU/mL. A similar reduction was observed for the 176z isolate, which declined from 2.24 × 10^4^ to 6.07 × 10^2^ CFU/mL under the same conditions.

In turn, the 186dz (*p* < 0.0001) and 205dz (*p* < 0.0001) isolates and Ye9N strain (*p* = 0.0029) became more resistant to NHS following RBC concentrate growth when compared to the control bacteria. For these isolates, an increase in CFU/mL was observed from 0 at 60 min to 3.24 × 10^6^, 4.7 × 10^3^, and 1.10 × 10^3^, respectively.

We observed no influence of RBC concentrate incubation on the bacterial serum sensitivity level for the 28s, 90z, 209z, and 58d isolates and Ye8 strain (*p* > 0.5, Figure 3, Table 2).

## 3. Discussion

RBC concentrate transfusion is a common and often life-saving medical procedure. However, RBC concentrate preparations are not routinely screened for microorganisms. While microbiological contamination of RBC concentrates is rare, it can result in severe or fatal outcomes for patients [4]. Enteropathogenic *Y. enterocolitica* contamination may originate from donors with bacteremia following intestinal yersiniosis [1]. There are numerous reports on the growth of *Y. enterocolitica* in RBC concentrates. However, the studied factors potentially impacting the growth of *Y. enterocolitica* in RBC concentrates include the process of blood preparation, RBC concentrate manufacturing method, use of additive solutions, blood group, donor sex, and time of blood exposure to room temperature [4,13,14,31,32,33]. The growth of different *Y. enterocolitica* biotypes in RBC concentrates has not been extensively studied.

The effect of temperature and temperature changes on *Y. enterocolitica* growth in RBC concentrates has previously been studied. Aplin et al. [31] observed rapid bacterial declines when RBC concentrate units were exposed to refrigeration and intermittent warming to 30 °C for 30 and 60 min. However, in studies applying constant storage temperatures (2–6 °C), *Y. enterocolitica* exhibited steady growth, reaching levels of 10^8^–10^9^ CFU/mL, consistent with our findings [2,4,13,14]. In our work, a constant temperature of incubation was applied to assess the development of *Y. enterocolitica* in standard conditions of RBC concentrate storage. Ramirez-Arcos et al. [4] demonstrated that variables related to RBC concentrate manufacturing, rather than the additive solution type or donor sex, had a greater impact on bacterial proliferation in RBC concentrate units. The same author also confirmed that exposure to uncontrolled temperature did not affect the growth of *Y. enterocolitica* in RBC concentrates [13]. Prax et al. [2] confirmed *Y. enterocolitica* as one of the key psychrotrophic bacteria capable of growing in refrigerated RBC concentrates, demonstrating its rapid and stable growth, reaching high bacterial concentrations (over 10^8^ CFU/mL) within 21 days. Only Graveman et al. [33] reported no *Y. enterocolitica* proliferation in RBC concentrates when whole blood (WB) units were spiked prior to component separation. However, their study primarily investigated the influence of whole blood manufacturing variables on bacterial proliferation, rather than the effects of prolonged incubation on bacterial growth. Our study further demonstrates that, in addition to manufacturing-related factors, intrinsic bacterial characteristics, such as biotype and genes for iron metabolism, significantly affect the growth dynamics of *Y. enterocolitica* in RBC concentrates. All isolates demonstrated growth in RBC concentrates over 28 days, with significant differences among bioserotypes. The highly pathogenic 1B/O:8 strain (Ye8) exhibited the highest proliferation, correlating with the presence of the yersiniobactin gene cluster (*ybtAESUX, irp1, irp2*) (Figure 1). Yersiniobactin, encoded on a pathogenicity island, seems to be crucial for bacterial development in RBC concentrates, playing a role as a siderophore-dependent uptake system for iron released from erythrocytes [17]. Strains lacking yersiniobactin showed lower growth potential, highlighting its importance. Interestingly, biotype 1A isolates, traditionally considered non-pathogenic, reached comparable counts and showed high allelic diversity in iron metabolism-related genes (Table 1, Figure 1). For example, the 28s strain, lacking the *hasA* heme acquisition gene [15], displayed reduced growth compared with other 1A isolates. This suggests that the ability to utilize heme as an iron source may be critical for optimal proliferation within the red blood cell environment for the *Y. enterocolitica* 1A biotype. Bioserotype 4/O:3 strains, characterized by a reduced repertoire of iron metabolism-related genes, including the absence of the *fepABCDG*, *fes*, *irp1*, *irp2*, *fyuA*, *hasR*, and *ybtAESUX* gene clusters, exhibited the lowest growth. This finding confirms that the lack of both enterochelin and yersiniabactin systems can severely limit bacterial proliferation, regardless of the biotype 4 pathogenic potential. Notably, isolates genetically similar to 4/O:3 but harboring additional iron metabolism genes (*fepBCDG, fes*), such as Ye9N and 58d, achieved higher growth, suggesting that gene content may outweigh genetic similarity in influencing RBC concentrate proliferation.

Serum resistance level is another factor influencing *Y. enterocolitica* survival in human blood [24]. Orozova et al. [14] reported that prolonged RBC concentrate incubation reduced the bacterial resistance to normal human serum (NHS). Our study directly examined the serum sensitivity post-RBC incubation, observing variable responses: increased resistance in three isolates including biotype 1A from wild boars (186dz and 205dz) and reference strain 2/O:9, decreased resistance in two isolates (4/O:3 3d and 176z), and no change in the remaining five isolates. The most NHS-resistant isolates belonged to biotype 1A, suggesting potential pathogenicity in strains historically considered non-pathogenic. This finding aligns with reports of wild boars as an emerging reservoir for human yersiniosis [30,34,35,36]. Notably, the 1A isolate 205dz, which carried the *ail* virulence gene and almost complete iron metabolism gene repertoire, supports reevaluating the pathogenic potential of certain 1A strains.

Our study faced limitations, including variability in donor ages, RBC concentrate bag volumes, and blood group types. However, previous research indicates that blood group (A, B, O) has minimal impact on *Y. enterocolitica* growth in RBC concentrates [14].

In conclusion, our findings demonstrate the capacity of diverse *Y. enterocolitica* bioserotypes to proliferate in red blood cells, influenced by iron metabolism-related genes. This emphasizes the clinical importance of *Y. enterocolitica* in RBC concentrate storage and highlights the need for prioritized microbiological testing when bacterial contamination is suspected. Continuous research and improved screening strategies are essential to ensure the microbiological safety of blood products. Furthermore, these findings highlight the need for a comprehensive reevaluation of the conventional biotype classification; although the biotype system may be retained, it should no longer be used as a definitive criterion for determining pathogenic potential.

## 4. Materials and Methods

### 4.1. Bacterial Strains

Eight *Y. enterocolitica* isolates and two *Y. enterocolitica* reference strains were included in this study. Table 3 contains the preliminary characteristics of the eight *Y. enterocolitica* isolates and *Y. enterocolitica* reference strains used in this study [37].

### 4.2. Detection of Virulence-Associated Genes of Y. enterocolitica

PCR was used to detect virulence-associated genes: *irp2*, *ystA*, *16S*, *ail*, *ystB*, *ymoA*, *yadA*, *ystC*, *irp1*, *ureC*, *virF*, *invA*, *yst*, *blaA*, *blab*, *myfA*, *myfB*, *myfC*, *chiY*, *ysrS*, *yst1M*, *sat*, *hreP*, *fepA*, *fepD*, *rfbC*, *tccC*, *fyuA*. The PCR mixtures consisted of 1 mM MgCl_2_, 1 × buffer, 0.2 mMdNTP, primers (Genomed, Warszawa, Poland), used at concentration ranging from 0.15 to 1.25 µM (Supplementary Table S2), 1U of *Taq* DNA polymerase, and 1 µL of DNA template. PCRs were performed with an initial denaturation step at 94 °C for 3 min, 35 cycles each of denaturation, annealing and extension, as indicated in Supplementary Table S2, and a final extension of 10 min at 72 °C. Amplicons were separated in 1.5% agarose gel containing 0.5 μg/mL ethidium bromide, at 120–130 V for 1 h and documented using the GelDocXR System (Bio-Rad, Hercules, CA, USA) [38,39].

### 4.3. Sequencing of the Genomes

Eight *Y. enterocolitica* isolates were subjected to whole genome sequencing (WGS). The DNA for the genomic sequencing was extracted with the MasterPure™ Complete DNA and RNA Purification Kit (Lucigen, Middleton, WI, USA) according to the manufacturer’s instructions. De novo whole-genome was performed on an Illumina MiSeq sequencer using the Nextera XT Library Preparation Kit with standard protocols. Libraries were sequenced using 2 × 300 bp paired-end v3 Reagent Kit (Illumina), following the manufacturer’s protocols [40]. The reads were trimmed and assembled into contigs using Shovill software, version 0.9.0 (https://github.com/tseemann/shovill, accessed on 12 June 2025). The quality of the obtained sequences was checked using the Quast program [41]. Genomes of publicly available *Y. enterocolitica* reference strains were included in the study—Ye9N and Ye8 (8081) (accession numbers: JAALCX000000000 and AM286415, respectively).

### 4.4. Phylogenetic Analysis of the Isolates and Iron Metabolism Gene Detection

Nucleotide sequences of 42 genes responsible for iron metabolism were obtained from GenBank, accession numbers AM286415 and U41370. Their detection in the genomes was performed using the Genome Comparator module (version 2.8.5) in BIGSdb [42] with gene presence defined as a BLAST (version 1.6.3) match of sequence identity >70% over 50% of a locus length. cgMLST was performed according to Savin et al. [43] using the BIGSdb—*Yersinia* database at https://bigsdb.pasteur.fr/yersinia (accessed on 12 June 2025), employing the *Yersinia* cgMLST scheme with standard settings. A phylogenetic tree was created using RapidNJ 2.3.2 software [44] using the Jukes and Cantor evolution model and visualized with Phandango [45].

### 4.5. Red Blood Cell Concentrate Units

The research material consisted of anonymized samples of RBC concentrate, obtained from healthy volunteer donors at the Regional Center for Blood Donation and Treatment in Wrocław, Poland. The RBC concentrate units used in this study are listed in Table 4. Donors consented to the use of their blood for scientific research when completing the qualification questionnaire before blood donation. This was conducted according to the principles expressed in the Declaration of Helsinki and was approved by the Director of Regional Center for Blood Donation and Treatment in Wrocław (cooperation agreement number N0BR000.7117.IN.6/Wet/2022). Donations of whole blood were taken into sterile, non-pyrogenic bags systems with anticoagulant CPD. After collection, to prevent bacterial growth, the whole blood donations were stored at room temperature (RT, 22 °C ± 2 °C) for at least 2 h, then centrifuged to obtain blood components including RBC concentrates in SAGM (saline-adenine-glucose-mannitol) solution. The first batch of whole blood was discarded thanks to the use of a pre-donation container. Each RBC concentrate used in the experiments was subjected to leukocyte depletion. Only RBC concentrates that met the specific criteria for acceptance of the blood donors in accordance with the national guidelines of the Ministry of Health in Poland, lacking hemolysis and negative for HIV 1/2, HCV and *Treponema pallidum* antibodies, a negative HBs antigen test, and negative viral genome screen (HIV RNA, HBV DNA, HCV RNA) were used [12,46]. The samples were not used for diagnostic tests, but were the research medium spiked with *Y. enterocolitica* isolates. Bacterial growth was observed under strictly defined conditions. The research used RBC concentrates after splitting into pediatric portions that had a shorter shelf life, (i.e., those that could not be transfused into adult recipients and were still an appropriate research model for planned experiments). All RBC concentrate units were tested for sterility before *Y. enterocolitica* infecting.

### 4.6. Incubation of Y. enterocolitica in Red Blood Cells

The study protocol was based on the method described by Prax et al., with modifications [2]. The volume of RBC concentrate in the bags, ranged from 50 to 167 mL. To avoid cross-contamination, the RBC concentrate volume was not normalized. The bacterial count was adjusted to the volume of RBC concentrate in the bag to obtain the starting bacterial count of 10^1^–10^2^ CFU/mL. For this, *Y. enterocolitica* was grown overnight in 5 mL of BHI broth (Th. Geyer Polska Sp. z o.o., Warszawa, Poland). Then, the cultures were diluted with sterile 0.85% saline solution to reach the OD_600_ of 0.1, corresponding to a bacterial count of 1.6–3.7 × 10^8^ CFU/mL, as determined by the plating of serial dilutions of bacteria. Next, the cultures were diluted 10,000 times, and the appropriate volume of bacterial suspension was taken to obtain the final bacterial count of 10^1^–10^2^ CFU/mL in a given RBC concentrate unit. Bacterial suspensions were transferred to RBC concentrate units using a sterile syringe needle through the sampling site coupler (Fenwal, Bloodbankdepot, Douglasville, GA, USA). Immediately after starting the incubation of bacteria in RBC concentrate units (time 0 days), 1 mL of RBC concentrate was taken from each bag and plated on solid media to determine the starting number of bacteria.

The RBC concentrate units were mixed by inversion and incubated at 2–6 °C with no agitation to mimic standard storage conditions. The bacterial counts in RBC concentrate were determined at days 3, 7, 14, 21, and 28. The RBC concentrates were sampled from the bags using a sampling site coupler under aseptic conditions. The serial RBC concentrate dilutions were plated onto BHI agar (Th. Geyer Polska Sp. z o.o., Warszawa, Poland) in triplicate, then incubated at 25 °C for 48 h.

### 4.7. Normal Human Serum Bactericidal Tests

The NHS bactericidal assay was carried out with major modifications according to Orozova et al. [14]. At least 50 mL of RBC concentrate was transferred to sterile tubes and centrifuged at 770× *g* for 9 min. Supernatants were pipetted into fresh 15 mL sterile tubes, then subsequently centrifuged at 4000 rpm, 4 °C, 20 min; after that, the supernatants were discarded. The bacterial pellets were then suspended in 5 mL of BHI broth. The density of bacterial suspensions was adjusted to an OD_600_ value equal to 0.8–0.9 (Spark^®^ Microplate Reader, Tecan, Männedorf, Switzerland). These optimized suspensions were then centrifuged again (4000 rpm, 4 °C, 20 min). The pellets were then suspended in 3 mL of NaCl (Th. Geyer Polska Sp. z o.o., Warszawa, Poland). One mL of these bacterial mixtures was refreshed in 5 mL of 0.9% NaCl, and 1 mL of that was combined with 1 mL of NHS (Sigma-Aldrich, Darmstadt, Germany). Incubation with NHS lasted up to 1 h at 37 °C. The CFU/mL value was assessed at time points 0, 15, 30, and 60 min. For this, the mixture of bacteria in NHS were plated onto BHI agar, then incubated at 25 °C for 48 h and counted. The NHS bactericidal tests were also conducted using the same bacterial isolates without RBC concentrate incubation. The overnight *Y. enterocolitica* cultures were diluted using BHI broth to an OD_600_ 0.1 and then grown to reach an OD_600_ of 0.8–0.9. Subsequent stages of the test remained unchanged.

### 4.8. Statistical Methods

*Y. enterocolitica* growth in the RBC concentrate units was assessed in triplicate. Statistical analysis was performed with GraphPad Prism 9.1.1. The data from each NHS bactericidal assay and RBC incubation were compared by analysis of variance (two-way ANOVA) [24,47]. Bacterial survival rates were compared using Tukey’s multiple comparisons test. The *p*-values from these tests are shown in the text and the figures. A *p* < 0.05 indicates that the compared values were significantly different at a 95% confidence level.

## Figures and Tables

**Table 1:** Mean and SD of *Y. enterocolitica* growth in the RBC concentrate units in triplicates. The data from each RBC concentrate incubation were compared by analysis of variance (two-way ANOVA).

Days/Isolate	0		3		7		14		21		28	
	Mean	SD	Mean	SD	Mean	SD	Mean	SD	Mean	SD	Mean	SD
3d, human, 4/O:3	3.50 × 10^1^	3.12 × 10^1^	1.76 × 10^2^	2.01 × 10^2^	1.11 × 10^4^	1.91 × 10^4^	1.21 × 10^4^	1.76 × 10^4^	2.50 × 10^4^	1.19 × 10^4^	2.50 × 10^5^	4.81 × 10^4^
58d, human, 1B/O:8	2.00 × 10^1^	1.00 × 10^1^	2.42 × 10^2^	3.97 × 10^2^	1.40 × 10^3^	2.15 × 10^3^	8.27 × 10^4^	5.85 × 10^4^	3.03 × 10^6^	2.16 × 10^6^	2.61 × 10^8^	1.32 × 10^8^
90z, pig, 4/O:3	2.53 × 10^1^	2.57 × 10^1^	3.77 × 10^1^	1.97 × 10^1^	1.88 × 10^2^	1.59 × 10^1^	4.22 × 10^3^	4.65 × 10^2^	2.21 × 10^4^	9.54 × 10^2^	6.05 × 10^5^	2.94 × 10^5^
176z, pig, 4/O:3	4.89 × 10^1^	2.17 × 10^1^	1.98 × 10^2^	2.15 × 10^2^	1.07 × 10^3^	1.29 × 10^3^	5.11 × 10^3^	2.71 × 10^3^	3.79 × 10^4^	1.75 × 10^4^	1.98 × 10^5^	8.91 × 10^4^
209z, pig, 1A	5.68 × 10^1^	2.01 × 10^1^	4.01 × 10^2^	2.91 × 10^2^	7.27 × 10^4^	1.25 × 10^5^	1.97 × 10^6^	2.98 × 10^6^	2.71 × 10^7^	1.77 × 10^7^	2.15 × 10^8^	2.69 × 10^7^
186dz, wild boar, 1A	7.00 × 10^1^	7.94 × 10^1^	9.83 × 10^1^	9.09 × 10^1^	1.17 × 10^3^	1.01 × 10^3^	7.13 × 10^5^	9.07 × 10^5^	6.55 × 10^6^	2.49 × 10^6^	2.67 × 10^8^	8.42 × 10^7^
205dz, wild boar, 1A	5.67 × 10^1^	4.17 × 10^1^	4.26 × 10^2^	6.70 × 10^2^	4.42 × 10^4^	7.34 × 10^4^	1.45 × 10^6^	2.13 × 10^6^	1.49 × 10^7^	3.20 × 10^6^	4.47 × 10^8^	2.52 × 10^8^
28s, roe deer, 1A	4.67 × 10^1^	1.26 × 10^1^	1.41 × 10^2^	1.58 × 10^2^	1.12 × 10^4^	1.89 × 10^4^	8.10 × 10^4^	1.02 × 10^5^	2.04 × 10^6^	1.03 × 10^6^	3.41 × 10^6^	8.02 × 10^5^
Ye9N, reference strain 2/O:9	4.00 × 10^1^	3.97 × 10^1^	3.79 × 10^2^	6.07 × 10^2^	2.14 × 10^4^	3.56 × 10^4^	4.91 × 10^5^	7.96 × 10^5^	9.22 × 10^8^	1.20 × 10^9^	5.69 × 10^8^	8.78 × 10^8^
Ye8, reference strain 1B/O:8	3.00 × 10^1^	2.00 × 10^1^	1.43 × 10^2^	1.16 × 10^2^	8.49 × 10^3^	1.09 × 10^4^	7.76 × 10^7^	1.30 × 10^8^	1.24 × 10^9^	1.14 × 10^9^	1.20 × 10^9^	1.01 × 10^9^

**Table 2:** Bacterial serum survival before and after 28 days of RBC concentrate incubation. The data from each tests were compared by analysis of variance (two-way ANOVA).

Isolate/ Time [min]	Before RBC		After RBC		Before RBC		After RBC		Before RBC		After RBC		Before RBC		After RBC	
	0				15				30				60			
	Mean	SD	Mean	SD	Mean	SD	Mean	SD	Mean	SD	Mean	SD	Mean	SD	Mean	SD
3d	5.07 × 10^5^	3.33 × 10^5^	7.60 × 10^5^	1.16 × 10^6^	2.77 × 10^5^	1.55 × 10^5^	1.18 × 10^5^	1.30 × 10^5^	2.31 × 10^5^	1.05 × 10^5^	2.89 × 10^3^	1.65 × 10^3^	2.14 × 10^5^	9.83 × 10^4^	1.10 × 10^2^	1.65 × 10^2^
58d	5.15 × 10^7^	1.18 × 10^5^	1.09 × 10^8^	6.41 × 10^7^	3.95 × 10^6^	2.52 × 10^6^	4.29 × 10^4^	3.19 × 10^3^	2.21 × 10^6^	1.79 × 10^6^	9.50 × 10^3^	7.47 × 10^3^	9.13 × 10^5^	1.07 × 10^6^	3.33 × 10^4^	2.82 × 10^4^
90z	7.58 × 10^4^	8.08 × 10^3^	4.44 × 10^5^	7.59 × 10^5^	3.79 × 10^4^	2.41 × 10^4^	1.49 × 10^4^	1.10 × 10^4^	0	0	3.00 × 10^3^	2.18 × 10^3^	0	0	3.67 × 10^1^	5.51 × 10^1^
176z	5.26 × 10^5^	9.97 × 10^4^	3.71 × 10^5^	2.53 × 10^5^	2.80 × 10^5^	2.16 × 10^4^	8.15 × 10^4^	5.96 × 10^4^	2.59 × 10^5^	1.96 × 10^4^	8.60 × 10^3^	1.27 × 10^4^	2.24 × 10^4^	7.37 × 10^3^	6.07 × 10^2^	1.03 × 10^3^
209z	2.48 × 10^7^	1.06 × 10^7^	2.23 × 10^7^	3.68 × 10^7^	3.33 × 10^2^	5.77 × 10^2^	6.67 × 10^2^	1.15 × 10^3^	0	0	6.67 × 10^2^	1.15 × 10^3^	0	0	3.77 × 10^2^	5.41 × 10^2^
186dz	3.36 × 10^7^	1.59 × 10^7^	3.28 × 10^7^	6.10 × 10^6^	1.17 × 10^3^	1.26 × 10^3^	3.54 × 10^7^	1.37 × 10^7^	0	0	2.48 × 10^7^	2.31 × 10^7^	0	0	3.24 × 10^6^	3.02 × 10^6^
205dz	2.22 × 10^7^	1.69 × 10^7^	6.43 × 10^7^	1.24 × 10^7^	6.67 × 10^2^	1.15 × 10^3^	2.09 × 10^4^	1.78 × 10^4^	0	0	2.86 × 10^4^	2.49 × 10^4^	0	0	4.70 × 10^3^	3.75 × 10^3^
28s	1.98 × 10^7^	1.66 × 10^7^	1.31 × 10^6^	1.35 × 10^6^	0	0	6.67 × 10^2^	5.77 × 10^2^	0	0	3.33 × 10^2^	5.77 × 10^2^	0	0	1.33 × 10^1^	2.31 × 10^1^
Ye8	1.55 × 10^7^	1.25 × 10^7^	1.62 × 10^7^	1.39 × 10^7^	9.36 × 10^6^	7.48 × 10^6^	2.34 × 10^3^	4.74 × 10^2^	1.73 × 10^5^	9.96 × 10^4^	2.00 × 10^3^	1.41 × 10^3^	4.52 × 10^4^	2.29 × 10^4^	3.68 × 10^3^	9.55 × 10^2^
Ye9N	1.35 × 10^7^	8.03 × 10^6^	1.84 × 10^7^	2.42 × 10^7^	3.09 × 10^4^	1.61 × 10^4^	1.79 × 10^4^	1.39 × 10^4^	1.56 × 10^3^	5.10 × 10^2^	4.67 × 10^3^	4.73 × 10^3^	0	0	1.10 × 10^3^	8.06 × 10^2^

**Table 3:** Characteristics of *Y. enterocolitica*.

*Y. enterocolitica*	Origin	Bioserotype	Virulence-Associated Genes Content
3d	Human feces	4/O:3	*yadA, ail, inv, yst, ystA, ystC, myfA, myfB, ureC, ymoA, rfbC, blaA, blaB*
58d		1B/O:8	*yadA, ail, inv, yst, ystA, ystC, myfA, myfB, ureC, ymoA, rfbC, fepD, blaA, blaB*
90z	Pigs	4/O:3	*yadA, ail, inv, yst, ystA, ystC, myfA, myfB, ureC, ymoA, rfbC, blaA, blaB*
176z		4/O:3	*yadA, ail, inv, yst, ystA, ystC, myfA, myfB, ureC, ymoA, rfbC, hreP, sat, blaA, blaB*
209z		1A/O:9	*inv, ystB, ureC, ymoA, fepD*
186dz	Wild boars	1A/NT	*inv, ureC, ymoA, fepA, fepD*
205dz		1A/NT	*ail, inv, ystB, myfA, ureC, ymoA, hreP, fepD*
28s	Roe deer	1A/NT	*inv, ystB, ureC, ymoA, tccC, hreP, fepA, fepD*
Ye9N	Reference	2/O:9	*yadA, virF, ail, inv, yst, ystA, ystC, myfA, myfB, ureC, ymoA, tccC, hreP, fepD, blaA, blaB*
Ye8		1B/O:8	*yadA, ail, inv, yst, ystA, ystC, myfA, myfB, ureC, ymoA, irp1, irp2, fyuA, chiY, Yts1M, hreP, fepD, blaB, ysrS*

NT—lack of serotype information.

**Table 4:** List of RBC concentrate units used in this study. SAGM—saline-adenine-glucose-mannitol; 0.2 J denotes a 0.2 portion of the whole RBC concentrate component for adults, which has been divided into smaller parts for pediatric use.

No.	Strain	Blood Group	Characteristics of RBC Concentrate Units	Volume [mL]	Age of RBC Concentrate on the Infecting Day
1	3d	0 RhD+	SAGM/450 mL/2-6C, leukoreduced, 0.2 J	50	31
2		0 RhD+	SAGM/450 mL/2-6C, irradiated, leukoreduced, 0.4 J	84	7
3		B RhD+	SAGM/450 mL/2-6C, irradiated, leukoreduced, 0.4 J	100	9
4	58d	A RhD+	SAGM/450 mL/2-6C, leukoreduced, 0.2 J	50	27
5		A RhD-	SAGM/450 mL/2-6C, leukoreduced, 0.4 J	93	25
6		A RhD+	SAGM/450 mL/2-6C, leukoreduced, 0.4 J	103	13
7	90z	A RhD+	SAGM/450 mL/2-6C, leukoreduced, 0.6 J	128	25
8		A RhD+	SAGM/450 mL/2-6C, leukoreduced, 0.6 J	139	21
9		0 RhD-	SAGM/450 mL/2-6C, leukoreduced, 0.6 J	119	12
10	176dz	0 RhD+	SAGM/450 mL/2-6C, leukoreduced, 0.8 J	167	22
11		B RhD+	SAGM/450 mL/2-6C, leukoreduced, 0.4 J	100	12
12		B RhD+	SAGM/450 mL/2-6C, leukoreduced, 0.4 J	105	21
13	209z	A RhD+	SAGM/450 mL/2-6C, leukoreduced, 0.4 J	65	23
14		A RhD-	SAGM/450 mL/2-6C, leukoreduced, 0.4 J	93	25
15		0 RhD+	SAGM/450 mL/2-6C, leukoreduced, 0.4 J	116	28
16	186dz	B RhD+	SAGM/450 mL/2-6C, leukoreduced, 0.4 J	86	18
17		A RhD+	SAGM/450 mL/2-6C, leukoreduced, 0.6 J	142	19
18		AB RhD+	SAGM/450 mL/2-6C, leukoreduced, 0.4 J	100	22
19	205dz	0 RhD+	SAGM/450 mL/2-6C leukoreduced, 0.6 J	140	25
20		A RhD-	SAGM/450 mL/2-6C, leukoreduced, 0.4 J	82	25
21		0 RhD-	SAGM/450 mL/2-6C, leukoreduced, 0.4 J	101	27
22	28s	B RhD+	SAGM/450mL/2-6C, leukoreduced, 0.6 J	154	29
23		A RhD-	SAGM/450 mL/2-6C, leukoreduced, 0.6 J	134	15
24		A RhD-	SAGM/450 mL/2-6C, leukoreduced, 0.6 J	135	20
25	Ye9N	B RhD+	SAGM/450 mL/2-6C, leukoreduced, 0.4 J	71	23
26		0 RhD+	SAGM/450 mL/2-6C, leukoreduced, 0.6 J	150	23
27		0 RhD+	SAGM/450 mL/2-6C, leukoreduced, 0.6 J	150	22
28	Ye8	0 RhD+	SAGM/450 mL/2-6C, leukoreduced, 0.4 J	88	29
29		B RhD+	SAGM/450 mL/2-6C, leukoreduced, 0.4 J	65	18
30		B RhD+	SAGM/450 mL/2-6C, leukoreduced, 0.4 J	118	21

## Data Availability

The original contributions presented in this study are included in the article and Supplementary Material. Further inquiries can be directed to the corresponding author.

## Supplementary Materials

The following supporting information can be downloaded at: https://www.mdpi.com/article/10.3390/ijms26125775/s1. References [48,49,50,51,52,53,54,55,56] are cited in the supplementary materials.
