# Supplementary material for: Biotype Determines Survival of Yersinia enterocolitica in Red Blood Cell Concentrates
Source: Int J Mol Sci. 2025 Jun 16;26(12):5775. doi: 10.3390/ijms26125775 (PMC12193566; doi:10.3390/ijms26125775)
Supplement: Supplementary file 1 [file ijms-26-05775-s001.zip › Supplementary Table S2.docx]

Supplementary Table S2. Primers used in PCR detection of virulence-associated genes in Yersinia spp. isolates.

| **Gene** | **Gene product/ function** | **Primers sequence (5'→3')** | **Primers concentration [µM]** | **Amplicon size**  **[bp]** | **PCR conditions (°C, s)** | | | |
| --- | --- | --- | --- | --- | --- | --- | --- | --- |
|  |  |  |  |  | **Denaturation** | **Annealing** | **Extension** | **Reference** |
| 16S rDNA | 16S rDNA gene of *Y. enterocolitica* | AATACCGCATAACGTCTTCG  CTTCTTCTGCGAGTAACGTC | 0.5 | 330 | 94, 45 | 59, 45 | 72, 45 | [48] |
| *yadA* | Marker for the presence of virulence plasmid pYV. Complement resistance | TAAGATCAGTGTCTCTGCGGCA  TAGTTATTTGCGATCCCTAGCAC | 0.15 | 747 | 94, 60 | 58, 90 | 72, 90 | [49] |
| *virF* | Transcriptional activator | CATGGCAGAACAGCAGTCAG  ACTCATCTTACCATTAAGAAG | 0.15 | 590 | 94, 60 | 58, 90 | 72, 90 | [49] |
| *ail* | Adhesive-invasive protein of pathogenic biotypes of *Y. enterocolitica* | TAGTTCTCTAATAGCCTGTTTATC  ACTATCTGAGATGATTAGAATCG | 0.15 | 531 | 94, 45 | 50, 60 | 72, 45 | [50] |
| *yst* | Enterotoxin | AATGCTGTCTTCATTTGGAGC  GCAACATACATCACAGCAATC | 0.15 | 163 | 94, 60 | 58, 90 | 72, 90 | [51] |
| *ystA* | Enterotoxin YstIA of pathogenic biotypes (1B, 2-5) | GTCTTCATTTGGAGGATTCGGC  AATCACTACTGACTTCGGCTGG | 1.25 | 134 | 94, 45 | 54, 45 | 72, 45 | [50] |
| *ystB* | Enterotoxin YstIB of nonpathogenic biotype 1A, that control the production of heat stable enterotoxins | TGTCAGCATTTATTCTCAACT  GCCGATAATGTATCATCAAG | 0.4 | 180 | 94, 45 | 50, 60 | 72, 45 | [50,52] |
| *ystC* | Enterotoxin YstIC of nonpathogenic biotype 1A that control the production of heat stable enterotoxins | TCGACAAGTGAGTGACGGAG  CCCTTACTCGCGACGAAATA | 0.2 | 284 | 94, 60 | 58, 90 | 72, 90 | [50,52] |
| *ysrS* | Chromosomal secretion III system termed Ysa | GCTCCTCATTACATAAATCGG  ATTCTCTCGTACAGATAGCG | 0.2 | 618 | 94, 45 | 58, 45 | 72, 45 | [53] |
| *myfA* | The basic component of the Myf antigen involved in the colonization of the intestine | CAGATACACCTGCCTTCCATCT  CTCGACATATTCCTCAACACGC | 0.2 | 272 | 94, 45 | 58, 45 | 72, 45 | [53] |
| *myfB* | Specific periplasmic protein responsible for the proper architecture of the Myf antigen | AAGTTTTCAGTGAGGACTGG  CTGTTGTCCATTACGGTGCC | 0.2 | 458 | 94, 45 | 58, 45 | 72, 45 | [53] |
| *myfC* | Outer membrane protein responsible for the proper architecture of the Myf antigen | CTGAATCTCAATTGGTCGCG  GTAAATCGGTAGTTTCCAGC | 0.2 | 672 | 94, 45 | 58, 45 | 72, 45 | [53] |
| *irp1* | Yersiniabactin synthetase HMWP, characteristic of the epidemic strain 1B/O:8 | GTACAGACCGCCTGCTCCAGTT  TGTAACCTACCTGCCTGTCGTC | 0.15 | 412 | 94, 60 | 58, 90 | 72, 90 | [53] |
| *irp2* | Yersiniabactin HMWP2, characteristic of the epidemic strain 1B/O:8 | CTCCGCAGAACAGGTAGCCGA  CGACATACTCAATCTGTCCGG | 1.25 | 500 | 94, 45 | 58, 45 | 72, 45 | [53] |
| *fyuA* | The receptor responsible for taking siderophore with Fe^3+^ | CTACGACATGCCGACAATGCC  TGCTTCCCGCGCCATAACGTG | 0.2 | 650 | 94, 30 | 59, 45 | 72, 45 | [53] |
| *Yts1M* | Component of the *Yersinia* chromosomal type II secretion systems termed Yts1 | ACACAAAACCTACACAGCGC  CAACGTGGTGATATGAACCC | 0.2 | 324 | 94, 45 | 58, 45 | 72, 45 | [53] |
| *chiY* | Chromosomal gene coding secretion substrat in Yts1 type II secretion system of *Y. enterocolitica*, putative chitin-binding protein | CGATTCATTAGATCTGACGC  TCGAAATGAATAGCCAGTGC | 0.2 | 618 | 94, 45 | 58, 45 | 72, 45 | [53] |
| *inv* | Invasin | TGCCTTGGTATGACTCTGCTTCA  AGCGCACCATTACTGGTGGTTAT | 0.15 | 1140 | 94, 60 | 58, 90 | 72, 90 | [54] |
| *tccC* | Insecticidal toxin complex-like protein | GGGCAAAAAATGCGTGAAGAGAG  TTTACCGGAATAACGCACAGTTTTA | 0.24 | 1035 | 94, 30 | 59, 60 | 72, 60 | [54] |
| *hreP* | Subtilisin/kexin-like protease | GCCGCTATGGTGCCTCTGGTGTG  CCCGCATTGACTCGCCCGTATC | 0.24 | 757 | 94, 45 | 69, 45 | 72, 45 | [54] |
| *fepA* | Enterochelin receptor protein | TACGCCAAAATACCTTACGAT TGTAAATACACCCCCACCTGA | 0.24 | 438 | 94, 30 | 54, 60 | 72, 60 | [54] |
| *fepD* | Enterochelin ABC transporter | GTGTGATTGCCTTACTATTG CGGTCATCCTTTTATTACGG | 0.24 | 381 | 94, 30 | 54, 60 | 72, 60 | [54] |
| *sat* | Streptogramin acetyltransferase | CCGATGGTGGGGTTTTCTCAAG  GGGATTACCGCCGACCACACTA | 0.24 | 456 | 94, 45 | 69, 45 | 72, 45 | [54] |
| *blaA* | Class A constitutive broad spectrum penicillinase | AAATGCGCTACCGGCTTCAG  AGTGGTGGTATCACGTGGGT | 0.1 | 439 | 95, 30 | 56, 30 | 72, 90 | [55] |
| *blaB* | Class C inducible cephalosporinase (AmpC) | CCCACTTTATACCTTGGCACAAA  GAACATATCTCCTGCCTGGGAAAT | 0.1 | 827 | 95, 30 | 56, 30 | 72, 90 | [55] |
| *rfbC* | Marker characteristic for *Y. enterocolitica* serotype O:3 | CGCATCTGGGACACTAATTCG  CCACGAATTCCATCAAAACCACC | 0.24 | 405 | 94, 30 | 59, 60 | 72, 60 | [56] |
| *ureC* | Urease | CTGCGTGGATATGGTGAAGAGT  CATTAGAGAGCGCATGGTAAGC | 0.15 | 358 | 94, 60 | 58, 90 | 72, 90 | [57] |
| *ymoA* | *Yersinia* modulator, negative regulator of genes *yop*, *virF* and *inv* | GACTTTTCTCAGGGGAATAC  GCTCAACGTTGTGTGTCT | 0.15 | 330 | 94, 45 | 50, 60 | 72, 45 | [52] |
